# 

**Published:** 1878-03

**Authors:** 


### MICHIGAN DENTAL ASSOCIATION.

The twenty-third annual session of the Michigan Dental Association will be held at Cook's Hotel, Ann Arbor, on the 26th, 27th and 28th of March, 1878, commencing at two o'clock, p. m., on Tuesday, March 26th. It is hoped that every member of the association will be present during the whole session.

All who desire to become members are requested to make application on the first day.

A general invitation is hereby given to all dentists doing business in Michigan, or anywhere else to attend.

The standing committees to report papers for consideration during the session are:

On Anatomy and Histology, D. C. Hawxhurst, D. D. S., M. D., Battle Creek; W. H. Jackson, D. D. S., and G. W. Stone, M. D. Title of paper not given.

On Physiology: W. G. Stowell, D. D. S., Manchester; F.

W. Clawson, D. D. S., and Thomas R. Perry. Title of paper, Saliva.

On Chemistry and Materia Medica: G. S. Shattuck, D. D. S., Detroit; G. E. Corbin, M. D., and J. E. Post, D. D. S. Title of paper, The Chemistry of the Oral Secretions.

On Hygiene: Lloyd L. Davis, D. D. S., Eaton Rapids; E. G. Douglass and W. D. Tremper, D. D. S. Title of paper not named.

On Pathology and Semeiology: Prof. A. J. Watling, D. D. S., Ypsilanti; R. H. Tremper, D. D. S., and J. W. Finch. Title of paper not named.

On Therapeutics: Prof. J. Taft, D. D. S., Ann Arbor; Isaac Douglass. D. D. S., and A. T. Metcalf, D. D. S. Title of paper, Action and Uses of Medicines in Dental Practice.

On Surgery, embracing the treatment and cure of all the diseased conditions of the teeth and their dependencies, either wholly or in part by manual operations. E. C. Moore, D. D. S., Detroit; George L. Field, D. D. S., and E. Hunter. Title of paper, Thoughts on Filling Teeth.

On Dental Education and Etiquette: E. S. Holmes, D. D. S., Grand Rapids; W. H. Dorrance and C. L. Vaughan, D. D. S. Title of paper, Is Dentistry a Profession or a Trade? and the education necessary to prepare one for its pursuit.

Executive Committee: C. B. Porter, Ann Arbor; J. A. Harris and J. A. Robinson.

Board of Censors: D. C. Hawxhurst, D. D. S., M. D., Battle Creek; W. H. Jackson, D. D. S. and Lloyd L. Davis, D. D. S.

Dental Department Advisory Committee: A. T. Metcalf, D. D. S., Kalamazoo; B. T. Spellman and G. R. Thomas D. D. S.

### DENTAL LAW.

The dentists of Mississippi are making an effort for the procurement of a law to regulate the practice of dentistry in that state, and to that end they now have a bill pending in the legislature. We hope they may soon realize their desires in that respect. From some experience in the same kind of work we would suggest that the object can not be obtained without well directed and persistent effort.

### CINCINNATI BI-ENNIAL MUSICAL FESTIVAL

Will be held May 14, 15, 16 and 17, 1878. The musical direction will be in charge of Theodore Thomas, assisted by Otto Singer. These gentlemen are equal, if not superior, to any in this country for the conduct of such an enterprise.

The following Soloists have been secured, viz.: *Soprano* Mdme. Eugenie Pappenheim, Mrs. E. Aline Osgood. *Tenor*, Mr. Charles Adams, Mr. Christian Fritsch. *Baritone*, Sig. G. Tagliapietra. *Contralto*, Miss Annie Louise Cary, Miss Emma Cranch, Miss Louise Rollwagen. *Bass*, Mr. M. R. Whitney, Mr. Franz Remmertz. Organist Geo E. Whitney, Thus making in this direction a presentation of rare excellence.

In addition to those mentioned the renowned Thomas Orchestra will be increased, for the occasion, to one hundred performers by the addition of the New York Philharmonic society and distinguished Soloists. This will undoubtedly be the most perfect Festival Orchestra in the world.

The Mass Chorus is composed of singers carefully selected for their voices and power of execution. These selections have been made from a large number of Choral societies of note and prominence.

The great organ used on the occasion is one of the five great organs in the world, and will upon this occasion be played for the first time. This organ is building in connection with, and is, in one sense, a part of the Grand Music Hall now nearing its completion.

This is the finest hall, in all its appointments, in this country, if not in the world.

The accumulated knowledge of the past has been made available in its construction, and the aim has been to make it most perfect in all respects.

The Festival of 1878 will have the distinguished honor of opening this magnificent Music Hall, which has been the gift to the people by Reuben R. Springer and his associates,

We have deemed it eminently proper thus to call attention to this great occasion, knowing that a large proportion of the readers of the REGISTER are greatly interested in music and all that pertains to it, and we hope that every reader of the REGISTER will have an opportunity on this occasion of hearing some of the grandest music in the world, and of seeing a marvel, in the way of a musical instrument; and a magnificent building.

Mr. Geo. Ward Nichols is President of the Board of Directors.

Mr. W. W. Taylor, Secretary.

Mr. John Shillito, Treasurer.

*THE DENTAL REGISTER,*

A MONTHLY MAGAZINE DEVOTED TO THE INTERESTS OF

THE DENTAL PROFESSION.

*Terms Reduced to $2.50 per Year. Single Copies 25c.*

*EDITED BY PROF. J. TAFT, D. D. S.,*

AND PUBLISHED BY

SPENCER & CROCKER, Ohio Dental Depot.

The volume commences in January and closes in December.

The REGISTER being issued monthly is always fresh, therefore Indispensable to the practitioner who desires to keep posted up with the new inventions and improvements which are daily developed. Neither expense nor effort will be spared to give value and variety to its contents. Articles will be illustrated with well executed engravings whenever they will add to the interest or assist to explanation.

Its extensive circulation and frequency of issue make it one of the BEST DENTAL ADVERTISING MEDIUMS in the United States.

All subscrptions must begin with January or July.

Local or special notices, five lines or less, will be inserted for $3.00 each insertion; over five lines, 50 cts. per line.

All advertising bills payable in advance, or at furthest, after the issue of the first number containing the advertisement. All transient advertisements to be paid for in advance.

☞CONTRIBUTIONS SOLICITED.

Exclusively Editorial Communications should be addressed to Editor.

The latest number of the REGISTER may be ordered with or without other goods at any time, and all letters should be addressed to

**SPENCER & CROCKER, Ohio Dental Depot, Cincinnati, O.**

